# Supplementary material for: Pyruvate Dehydrogenase Kinase 1 inhibition mediated oxidative phosphorylation enhancement in cartilage promotes osteoarthritis progression
Source: BMC Musculoskelet Disord. 2023 Jul 20;24:597. doi: 10.1186/s12891-023-06585-6 (PMC10357736; doi:10.1186/s12891-023-06585-6)
Supplement: Supplementary file 1 — Additional file 1. [file 12891_2023_6585_MOESM1_ESM.zip › BP.Gsea.1653623667859/GOBP_ENDOPLASMIC_RETICULUM_TO_GOLGI_VESICLE_MEDIATED_TRANSPORT.html]

Details for gene set GOBP\_ENDOPLASMIC\_RETICULUM\_TO\_GOLGI\_VESICLE\_MEDIATED\_TRANSPORT[GSEA]

|  || Dataset | 55457\_collapsed\_to\_symbols.cls.cls #OA\_versus\_normal.cls.cls #OA\_versus\_normal\_repos |
| Phenotype | cls.cls#OA\_versus\_normal\_repos |
| Upregulated in class | OA |
| GeneSet | GOBP\_ENDOPLASMIC\_RETICULUM\_TO\_GOLGI\_VESICLE\_MEDIATED\_TRANSPORT |
| Enrichment Score (ES) | 0.41775313 |
| Normalized Enrichment Score (NES) | 1.958914 |
| Nominal p-value | 0.0 |
| FDR q-value | 0.029036174 |
| FWER p-Value | 0.54 |
Table: GSEA Results Summary

  

Fig 1: Enrichment plot: GOBP\_ENDOPLASMIC\_RETICULUM\_TO\_GOLGI\_VESICLE\_MEDIATED\_TRANSPORT      
 Profile of the Running ES Score & Positions of GeneSet Members on the Rank Ordered List

  

| SYMBOL | TITLE | RANK IN GENE LIST | RANK METRIC SCORE | RUNNING ES | CORE ENRICHMENT || 1 | COPE | COPI coat complex subunit epsilon [Source:HGNC Symbol;Acc:HGNC:2234] | 15 | 1.136 | 0.0337 | Yes |
| 2 | KDELR3 | KDEL endoplasmic reticulum protein retention receptor 3 [Source:HGNC Symbol;Acc:HGNC:6306] | 77 | 0.950 | 0.0581 | Yes |
| 3 | PDCD6 | programmed cell death 6 [Source:HGNC Symbol;Acc:HGNC:8765] | 87 | 0.933 | 0.0861 | Yes |
| 4 | YIPF5 | Yip1 domain family member 5 [Source:HGNC Symbol;Acc:HGNC:24877] | 107 | 0.903 | 0.1123 | Yes |
| 5 | TMED3 | transmembrane p24 trafficking protein 3 [Source:HGNC Symbol;Acc:HGNC:28889] | 141 | 0.868 | 0.1364 | Yes |
| 6 | YIF1A | "Yip1 interacting factor homolog A, membrane trafficking protein [Source:HGNC Symbol;Acc:HGNC:16688]" | 145 | 0.867 | 0.1628 | Yes |
| 7 | ERGIC3 | ERGIC and golgi 3 [Source:HGNC Symbol;Acc:HGNC:15927] | 299 | 0.751 | 0.1739 | Yes |
| 8 | TRAPPC4 | trafficking protein particle complex subunit 4 [Source:HGNC Symbol;Acc:HGNC:19943] | 322 | 0.736 | 0.1947 | Yes |
| 9 | SEC13 | "SEC13 homolog, nuclear pore and COPII coat complex component [Source:HGNC Symbol;Acc:HGNC:10697]" | 460 | 0.677 | 0.2048 | Yes |
| 10 | LMAN2 | "lectin, mannose binding 2 [Source:HGNC Symbol;Acc:HGNC:16986]" | 485 | 0.666 | 0.2234 | Yes |
| 11 | COPB2 | COPI coat complex subunit beta 2 [Source:HGNC Symbol;Acc:HGNC:2232] | 504 | 0.658 | 0.2422 | Yes |
| 12 | LMAN2L | "lectin, mannose binding 2 like [Source:HGNC Symbol;Acc:HGNC:19263]" | 507 | 0.656 | 0.2622 | Yes |
| 13 | TRAPPC6A | trafficking protein particle complex subunit 6A [Source:HGNC Symbol;Acc:HGNC:23069] | 636 | 0.610 | 0.2709 | Yes |
| 14 | RANGRF | RAN guanine nucleotide release factor [Source:HGNC Symbol;Acc:HGNC:17679] | 660 | 0.603 | 0.2876 | Yes |
| 15 | TRAPPC2 | trafficking protein particle complex subunit 2 [Source:HGNC Symbol;Acc:HGNC:23068] | 914 | 0.538 | 0.2844 | Yes |
| 16 | TEX261 | testis expressed 261 [Source:HGNC Symbol;Acc:HGNC:30712] | 948 | 0.529 | 0.2980 | Yes |
| 17 | TRAPPC2L | trafficking protein particle complex subunit 2L [Source:HGNC Symbol;Acc:HGNC:30887] | 990 | 0.520 | 0.3108 | Yes |
| 18 | SEC22A | "SEC22 homolog A, vesicle trafficking protein [Source:HGNC Symbol;Acc:HGNC:20260]" | 1220 | 0.472 | 0.3074 | Yes |
| 19 | BET1 | Bet1 golgi vesicular membrane trafficking protein [Source:HGNC Symbol;Acc:HGNC:14562] | 1223 | 0.471 | 0.3217 | Yes |
| 20 | KDELR2 | KDEL endoplasmic reticulum protein retention receptor 2 [Source:HGNC Symbol;Acc:HGNC:6305] | 1486 | 0.431 | 0.3144 | Yes |
| 21 | SPAST | spastin [Source:HGNC Symbol;Acc:HGNC:11233] | 1502 | 0.429 | 0.3264 | Yes |
| 22 | BCAP29 | B cell receptor associated protein 29 [Source:HGNC Symbol;Acc:HGNC:24131] | 1548 | 0.421 | 0.3358 | Yes |
| 23 | BCAP31 | B cell receptor associated protein 31 [Source:HGNC Symbol;Acc:HGNC:16695] | 1584 | 0.416 | 0.3459 | Yes |
| 24 | RAB2A | "RAB2A, member RAS oncogene family [Source:HGNC Symbol;Acc:HGNC:9763]" | 1633 | 0.409 | 0.3547 | Yes |
| 25 | MPPE1 | metallophosphoesterase 1 [Source:HGNC Symbol;Acc:HGNC:15988] | 1675 | 0.402 | 0.3638 | Yes |
| 26 | USE1 | unconventional SNARE in the ER 1 [Source:HGNC Symbol;Acc:HGNC:30882] | 1723 | 0.395 | 0.3722 | Yes |
| 27 | PEF1 | penta-EF-hand domain containing 1 [Source:HGNC Symbol;Acc:HGNC:30009] | 1784 | 0.387 | 0.3794 | Yes |
| 28 | TRAPPC12 | trafficking protein particle complex subunit 12 [Source:HGNC Symbol;Acc:HGNC:24284] | 1930 | 0.369 | 0.3794 | Yes |
| 29 | COPA | COPI coat complex subunit alpha [Source:HGNC Symbol;Acc:HGNC:2230] | 1959 | 0.366 | 0.3885 | Yes |
| 30 | ANK1 | ankyrin 1 [Source:HGNC Symbol;Acc:HGNC:492] | 2052 | 0.356 | 0.3922 | Yes |
| 31 | TMED1 | transmembrane p24 trafficking protein 1 [Source:HGNC Symbol;Acc:HGNC:17291] | 2212 | 0.337 | 0.3901 | Yes |
| 32 | KDELR1 | KDEL endoplasmic reticulum protein retention receptor 1 [Source:HGNC Symbol;Acc:HGNC:6304] | 2227 | 0.336 | 0.3993 | Yes |
| 33 | TMED7 | transmembrane p24 trafficking protein 7 [Source:HGNC Symbol;Acc:HGNC:24253] | 2256 | 0.331 | 0.4073 | Yes |
| 34 | RAB1B | "RAB1B, member RAS oncogene family [Source:HGNC Symbol;Acc:HGNC:18370]" | 2453 | 0.308 | 0.4015 | Yes |
| 35 | CTAGE9 | CTAGE family member 9 [Source:HGNC Symbol;Acc:HGNC:37275] | 2468 | 0.307 | 0.4098 | Yes |
| 36 | TRAPPC3 | trafficking protein particle complex subunit 3 [Source:HGNC Symbol;Acc:HGNC:19942] | 2527 | 0.300 | 0.4145 | Yes |
| 37 | TMED9 | transmembrane p24 trafficking protein 9 [Source:HGNC Symbol;Acc:HGNC:24878] | 2600 | 0.290 | 0.4178 | Yes |
| 38 | SAR1B | secretion associated Ras related GTPase 1B [Source:HGNC Symbol;Acc:HGNC:10535] | 2780 | 0.272 | 0.4121 | No |
| 39 | SCFD1 | sec1 family domain containing 1 [Source:HGNC Symbol;Acc:HGNC:20726] | 2935 | 0.257 | 0.4080 | No |
| 40 | RAB29 | "RAB29, member RAS oncogene family [Source:HGNC Symbol;Acc:HGNC:9789]" | 3020 | 0.247 | 0.4090 | No |
| 41 | VAMP7 | vesicle associated membrane protein 7 [Source:HGNC Symbol;Acc:HGNC:11486] | 3380 | 0.214 | 0.3875 | No |
| 42 | CUL3 | cullin 3 [Source:HGNC Symbol;Acc:HGNC:2553] | 3480 | 0.204 | 0.3860 | No |
| 43 | VCP | valosin containing protein [Source:HGNC Symbol;Acc:HGNC:12666] | 3518 | 0.201 | 0.3893 | No |
| 44 | MIA3 | MIA SH3 domain ER export factor 3 [Source:HGNC Symbol;Acc:HGNC:24008] | 3550 | 0.198 | 0.3930 | No |
| 45 | KLHL12 | kelch like family member 12 [Source:HGNC Symbol;Acc:HGNC:19360] | 3609 | 0.193 | 0.3943 | No |
| 46 | ZW10 | zw10 kinetochore protein [Source:HGNC Symbol;Acc:HGNC:13194] | 3643 | 0.190 | 0.3976 | No |
| 47 | TFG | trafficking from ER to golgi regulator [Source:HGNC Symbol;Acc:HGNC:11758] | 3719 | 0.183 | 0.3974 | No |
| 48 | GAS1 | growth arrest specific 1 [Source:HGNC Symbol;Acc:HGNC:4165] | 3757 | 0.181 | 0.4000 | No |
| 49 | SEC23B | "SEC23 homolog B, COPII coat complex component [Source:HGNC Symbol;Acc:HGNC:10702]" | 3838 | 0.172 | 0.3991 | No |
| 50 | SEC23IP | SEC23 interacting protein [Source:HGNC Symbol;Acc:HGNC:17018] | 3889 | 0.166 | 0.4002 | No |
| 51 | VAPB | VAMP associated protein B and C [Source:HGNC Symbol;Acc:HGNC:12649] | 3905 | 0.164 | 0.4041 | No |
| 52 | SEC24B | "SEC24 homolog B, COPII coat complex component [Source:HGNC Symbol;Acc:HGNC:10704]" | 3933 | 0.162 | 0.4069 | No |
| 53 | SEC23A | "SEC23 homolog A, COPII coat complex component [Source:HGNC Symbol;Acc:HGNC:10701]" | 4017 | 0.154 | 0.4052 | No |
| 54 | ATL2 | atlastin GTPase 2 [Source:HGNC Symbol;Acc:HGNC:24047] | 4025 | 0.154 | 0.4094 | No |
| 55 | ERGIC2 | ERGIC and golgi 2 [Source:HGNC Symbol;Acc:HGNC:30208] | 4126 | 0.144 | 0.4060 | No |
| 56 | YIPF4 | Yip1 domain family member 4 [Source:HGNC Symbol;Acc:HGNC:28145] | 4252 | 0.133 | 0.4003 | No |
| 57 | NRBP1 | nuclear receptor binding protein 1 [Source:HGNC Symbol;Acc:HGNC:7993] | 4423 | 0.119 | 0.3906 | No |
| 58 | CSNK1D | casein kinase 1 delta [Source:HGNC Symbol;Acc:HGNC:2452] | 4529 | 0.111 | 0.3858 | No |
| 59 | CTAGE1 | cutaneous T cell lymphoma-associated antigen 1 [Source:HGNC Symbol;Acc:HGNC:24346] | 4530 | 0.111 | 0.3892 | No |
| 60 | STX18 | syntaxin 18 [Source:HGNC Symbol;Acc:HGNC:15942] | 4968 | 0.075 | 0.3574 | No |
| 61 | USO1 | USO1 vesicle transport factor [Source:HGNC Symbol;Acc:HGNC:30904] | 4992 | 0.074 | 0.3578 | No |
| 62 | GOSR2 | golgi SNAP receptor complex member 2 [Source:HGNC Symbol;Acc:HGNC:4431] | 5037 | 0.070 | 0.3566 | No |
| 63 | TRIP11 | thyroid hormone receptor interactor 11 [Source:HGNC Symbol;Acc:HGNC:12305] | 5233 | 0.054 | 0.3430 | No |
| 64 | RNF139 | ring finger protein 139 [Source:HGNC Symbol;Acc:HGNC:17023] | 5636 | 0.021 | 0.3122 | No |
| 65 | TMED2 | transmembrane p24 trafficking protein 2 [Source:HGNC Symbol;Acc:HGNC:16996] | 5709 | 0.015 | 0.3070 | No |
| 66 | YKT6 | YKT6 v-SNARE homolog [Source:HGNC Symbol;Acc:HGNC:16959] | 5800 | 0.008 | 0.3002 | No |
| 67 | RINT1 | RAD50 interactor 1 [Source:HGNC Symbol;Acc:HGNC:21876] | 5863 | 0.004 | 0.2955 | No |
| 68 | SEC31A | "SEC31 homolog A, COPII coat complex component [Source:HGNC Symbol;Acc:HGNC:17052]" | 6021 | -0.006 | 0.2834 | No |
| 69 | PREB | prolactin regulatory element binding [Source:HGNC Symbol;Acc:HGNC:9356] | 6044 | -0.008 | 0.2820 | No |
| 70 | SORL1 | sortilin related receptor 1 [Source:HGNC Symbol;Acc:HGNC:11185] | 6143 | -0.016 | 0.2748 | No |
| 71 | GOSR1 | golgi SNAP receptor complex member 1 [Source:HGNC Symbol;Acc:HGNC:4430] | 6166 | -0.018 | 0.2736 | No |
| 72 | PPP6C | protein phosphatase 6 catalytic subunit [Source:HGNC Symbol;Acc:HGNC:9323] | 6195 | -0.020 | 0.2720 | No |
| 73 | SEC24D | "SEC24 homolog D, COPII coat complex component [Source:HGNC Symbol;Acc:HGNC:10706]" | 6335 | -0.031 | 0.2621 | No |
| 74 | LMAN1 | "lectin, mannose binding 1 [Source:HGNC Symbol;Acc:HGNC:6631]" | 6361 | -0.032 | 0.2612 | No |
| 75 | SEC24C | "SEC24 homolog C, COPII coat complex component [Source:HGNC Symbol;Acc:HGNC:10705]" | 6366 | -0.033 | 0.2618 | No |
| 76 | TRAPPC11 | trafficking protein particle complex subunit 11 [Source:HGNC Symbol;Acc:HGNC:25751] | 6423 | -0.037 | 0.2586 | No |
| 77 | HYOU1 | hypoxia up-regulated 1 [Source:HGNC Symbol;Acc:HGNC:16931] | 6775 | -0.061 | 0.2330 | No |
| 78 | ARF4 | ADP ribosylation factor 4 [Source:HGNC Symbol;Acc:HGNC:655] | 6894 | -0.072 | 0.2260 | No |
| 79 | STX5 | syntaxin 5 [Source:HGNC Symbol;Acc:HGNC:11440] | 6932 | -0.074 | 0.2254 | No |
| 80 | CREB3L2 | cAMP responsive element binding protein 3 like 2 [Source:HGNC Symbol;Acc:HGNC:23720] | 7580 | -0.125 | 0.1787 | No |
| 81 | TMED5 | transmembrane p24 trafficking protein 5 [Source:HGNC Symbol;Acc:HGNC:24251] | 7581 | -0.125 | 0.1825 | No |
| 82 | VAMP4 | vesicle associated membrane protein 4 [Source:HGNC Symbol;Acc:HGNC:12645] | 7608 | -0.128 | 0.1844 | No |
| 83 | SCAP | SREBF chaperone [Source:HGNC Symbol;Acc:HGNC:30634] | 7647 | -0.130 | 0.1855 | No |
| 84 | ARCN1 | archain 1 [Source:HGNC Symbol;Acc:HGNC:649] | 8112 | -0.164 | 0.1542 | No |
| 85 | COPG1 | COPI coat complex subunit gamma 1 [Source:HGNC Symbol;Acc:HGNC:2236] | 8289 | -0.178 | 0.1459 | No |
| 86 | WHAMMP3 | "WAS protein homolog associated with actin, golgi membranes and microtubules pseudogene 3 [Source:HGNC Symbol;Acc:HGNC:27892]" | 8350 | -0.182 | 0.1468 | No |
| 87 | CNIH4 | cornichon family AMPA receptor auxiliary protein 4 [Source:HGNC Symbol;Acc:HGNC:25013] | 8392 | -0.185 | 0.1493 | No |
| 88 | GBF1 | golgi brefeldin A resistant guanine nucleotide exchange factor 1 [Source:HGNC Symbol;Acc:HGNC:4181] | 8461 | -0.191 | 0.1498 | No |
| 89 | STX17 | syntaxin 17 [Source:HGNC Symbol;Acc:HGNC:11432] | 8466 | -0.191 | 0.1554 | No |
| 90 | SEC22B | "SEC22 homolog B, vesicle trafficking protein [Source:HGNC Symbol;Acc:HGNC:10700]" | 8614 | -0.202 | 0.1501 | No |
| 91 | VAPA | VAMP associated protein A [Source:HGNC Symbol;Acc:HGNC:12648] | 8854 | -0.221 | 0.1382 | No |
| 92 | SEC24A | "SEC24 homolog A, COPII coat complex component [Source:HGNC Symbol;Acc:HGNC:10703]" | 9357 | -0.260 | 0.1070 | No |
| 93 | PGAP1 | post-GPI attachment to proteins inositol deacylase 1 [Source:HGNC Symbol;Acc:HGNC:25712] | 9358 | -0.260 | 0.1149 | No |
| 94 | SEC16A | "SEC16 homolog A, endoplasmic reticulum export factor [Source:HGNC Symbol;Acc:HGNC:29006]" | 9434 | -0.268 | 0.1173 | No |
| 95 | COPB1 | COPI coat complex subunit beta 1 [Source:HGNC Symbol;Acc:HGNC:2231] | 9447 | -0.269 | 0.1246 | No |
| 96 | STEEP1 | STING1 ER exit protein 1 [Source:HGNC Symbol;Acc:HGNC:26239] | 9592 | -0.282 | 0.1220 | No |
| 97 | RAB1A | "RAB1A, member RAS oncogene family [Source:HGNC Symbol;Acc:HGNC:9758]" | 9604 | -0.284 | 0.1299 | No |
| 98 | CTAGE6 | CTAGE family member 6 [Source:HGNC Symbol;Acc:HGNC:28644] | 9691 | -0.290 | 0.1321 | No |
| 99 | YIF1B | "Yip1 interacting factor homolog B, membrane trafficking protein [Source:HGNC Symbol;Acc:HGNC:30511]" | 9960 | -0.317 | 0.1209 | No |
| 100 | TMED10 | transmembrane p24 trafficking protein 10 [Source:HGNC Symbol;Acc:HGNC:16998] | 9993 | -0.320 | 0.1282 | No |
| 101 | INSIG1 | insulin induced gene 1 [Source:HGNC Symbol;Acc:HGNC:6083] | 10264 | -0.347 | 0.1177 | No |
| 102 | GOLGA2 | golgin A2 [Source:HGNC Symbol;Acc:HGNC:4425] | 10285 | -0.349 | 0.1269 | No |
| 103 | TRAPPC8 | trafficking protein particle complex subunit 8 [Source:HGNC Symbol;Acc:HGNC:29169] | 10542 | -0.377 | 0.1185 | No |
| 104 | LMAN1L | "lectin, mannose binding 1 like [Source:HGNC Symbol;Acc:HGNC:6632]" | 11430 | -0.492 | 0.0643 | No |
| 105 | MIA2 | MIA SH3 domain ER export factor 2 [Source:HGNC Symbol;Acc:HGNC:18432] | 11652 | -0.525 | 0.0631 | No |
| 106 | SEC31B | "SEC31 homolog B, COPII coat complex component [Source:HGNC Symbol;Acc:HGNC:23197]" | 11690 | -0.531 | 0.0765 | No |
| 107 | SAR1A | secretion associated Ras related GTPase 1A [Source:HGNC Symbol;Acc:HGNC:10534] | 11962 | -0.586 | 0.0733 | No |
Table: GSEA details [plain text format]

  

Fig 2: GOBP\_ENDOPLASMIC\_RETICULUM\_TO\_GOLGI\_VESICLE\_MEDIATED\_TRANSPORT      
 Blue-Pink O' Gram in the Space of the Analyzed GeneSet

  

Fig 3: GOBP\_ENDOPLASMIC\_RETICULUM\_TO\_GOLGI\_VESICLE\_MEDIATED\_TRANSPORT: Random ES distribution      
 Gene set null distribution of ES for **GOBP\_ENDOPLASMIC\_RETICULUM\_TO\_GOLGI\_VESICLE\_MEDIATED\_TRANSPORT**
